# Laminin N-terminus α31 is upregulated in invasive ductal breast cancer and changes the mode of tumour invasion

**DOI:** 10.1371/journal.pone.0264430

**Published:** 2022-03-01

**Authors:** Lee D. Troughton, Danielle A. O’Loughlin, Tobias Zech, Kevin J. Hamill

**Affiliations:** 1 Cell and Molecular Physiology, Loyola University Chicago, Chicago, Illinois, United States of America; 2 Institute of Life Course and Medical Sciences, University of Liverpool, Liverpool, United Kingdom; 3 Institute of Systems, Molecular and Integrative Biology, University of Liverpool, Liverpool, United Kingdom; University of Alabama at Birmingham, UNITED STATES

## Abstract

Laminin N-terminus α31 (LaNt α31) is an alternative splice isoform derived from the laminin α3 gene. The LaNt α31 protein is enriched around the terminal duct lobular units in normal breast tissue. In the skin and cornea the protein influences epithelial cell migration and tissue remodelling. However, LaNt α31 has never been investigated in a tumour environment. Here we analysed LaNt α31 in invasive ductal carcinoma and determined its contribution to breast carcinoma invasion. LaNt α31 expression and distribution were analysed by immunohistochemistry in human breast tissue biopsy sections and tissue microarrays covering 232 breast cancer samples. This analysis revealed LaNt α31 to be upregulated in 56% of invasive ductal carcinoma specimens compared with matched normal tissue, and further increased in nodal metastasis compared with the tumour mass in 45% of samples. 65.8% of triple negative cases displayed medium to high LaNt α31 expression. To study LaNt α31 function, an adenoviral system was used to induce expression in MCF-7 and MDA-MB-231 cells. 2D cell migration and invasion into collagen hydrogels were not significantly different between LaNt α31 overexpressing cells and control treated cells. However, LaNt α31 overexpression reduced the proliferation rate of MCF-7 and MDA-MB-231 cells. Moreover, LaNt α31 overexpressing MDA-MB-231 cells displayed a striking change in their mode of invasion into laminin-containing Matrigel; changing from multicellular streaming to individual cellular-invasion. In agreement with these results, 66.7% of the tumours with the highest LaNt α31 expression were non-cohesive. Together these findings indicate that breast cancer-associated changes in LaNt α31 expression could contribute to the processes involved in tumour invasion and may represent a new therapeutic target.

## Introduction

An essential stage of tumour progression is acquisition of an ability to breakthrough an organised extracellular matrix (ECM) structure termed the basement membrane (BM) [1]. Determining the expression and distribution of BM proteins has yielded valuable biomarkers to predict breast cancer outcomes [2–6]. Much of this work has focused on the laminin (LM) family of BM proteins, which are not only essential barrier components, but also act as substrates for tumour cell migration, regulate actin dynamics, influence survival and growth signalling pathways, and maintain quiescence in cancer stem cell niches; all of which influence breast cancer progression [7–10]. Here we investigated a relatively unstudied LM-related protein, laminin N-terminus α31 (LaNt α31) that we predicted would change in cancer and which could therefore represent a new target for therapeutic development [11, 12].

LMs are obligate heterotrimeric proteins comprised of an α, β and γ chain, with each chain derived from one of five α genes (LAMA1-5), one of three β (LAMB1-3), and one of three γ (LAMC1-3), as reviewed in [7, 8, 13]. Through the use of distinct promoters, LAMA3 generates two structurally distinct LMs; a so-called “full-length” variant LMα3b, and the much shorter LMα3a [8, 13–15]. The LaNt proteins are also derived from LM-encoding genes, through intron-retention and polyadenylation within the retained intron [11]. Four LaNt family members have been identified at the transcript level; however, only LaNt α31, derived from the LAMA3 gene, has been confirmed at the protein level [11]. LaNt α31 displays widespread tissue distribution and is enriched in structured regions of ECM surrounding terminal duct lobular units (TDLUs) in normal breast tissue [11].

LaNt α31 functions have only been studied in corneal and skin epithelium to date, where upregulation of LaNt α31 was observed in response to corneal burn wounds or stem cells activation in ex vivo models, and where knockdown in expression reduced the rate at which epidermal keratinocytes close scratch wounds [11, 12]. Mechanistic studies have also indicated a role for this protein in modifying cell adhesion and migration via changes to matrix organisation and adhesion complex maturation [12, 16]. Further indications to LaNt α31 function come from its structure. Although LaNt α31 is smaller than LMs and lacks the coiled-coil domain required for LM trimer formation, it does share structural domains with LMα3b. Specifically, LaNt α31 is comprised of a LM N-terminal domain (LN domain) and two LM-type epidermal growth factor-like repeats (LE domains) [11]. LN domains are involved in LM-to-LM interaction, and therefore are essential for laminin network assembly in BMs [7, 17, 18]. LaNt α31 also contains 54 unique amino acids with no homology to known structural motifs but which have allowed specific antibodies to be raised against this protein [11, 12]. Intriguingly the LaNt α31 protein architecture is structurally similar to other members of the laminin superfamily family, the netrins. Netrins are predominantly known as signalling proteins; however, netrin-4, via its LN domain, can disrupt LM-LM interactions and change the structural characteristics of LM networks [19–21]. Moreover, netrin-4-induced changes to BM stiffness is a strong predictor of tumour metastasis and patient outcome in numerous cancers including breast cancer [22].

While functional data suggest that LaNt α31 could be capable of influencing tumour progression, further rationale for investigating this protein in tumour microenvironment comes from studies of the other, more comprehensively studied, products of the LAMA3 gene. Reduction of LMα3a and LMα3b in breast carcinoma has been independently reported by several groups [23–25], with LMα3b downregulation in tumour vasculature associated with later stage tumours [26]. However, the situation is more complicated than a simple linear relationship, as increased LMα3 has been associated with triple negative breast carcinoma, and increased immunoreactivity that correlated with tumour stage has also been reported with antibodies against conformational epitopes in LMα3β3γ2 (LM332), LMβ3 and LMγ2, the preferred trimerisation partners of LMα3a and LMα3b [25, 27].

Here we performed the first investigation into LaNt α31 in breast cancer. Breast tissue from normal and invasive ductal carcinomas were processed for immunohistochemistry with antibodies against LaNt α31, and correlations between staining intensity and pathology determined. LaNt α31 expression was upregulated in cultured breast carcinoma cells and the impact on cellular behaviour was determined. The results revealed that LaNt α31 is increased in tumour tissue, and is further increased in metastasis. Moreover, whereas induced LaNt α31 expression did not drive non-invasive breast cancer cells to become invasive, it did influence invasive breast cancer cells to change the mode of their invasion into LM-rich matrices. These findings Indicate that this little studied protein plays a role in defining how breast cancers disseminate.

## Methods

### Ethical approval

The Liverpool Bio-Innovation Hub Biobank conferred ethical approval in writing for the use of samples in this project (REC reference 14/NW/1212, NRES Committee North West–Haydock). Project specific ethical approval for working with human tissue was conferred in writing by the University of Liverpool Research Ethics Committees (approval number:7488). All archived patient tissues were collected with informed consent. Data were fully anonymised before granting access.

### Antibodies

Mouse monoclonal antibodies raised against human LaNt α31 were described previously and were used at 0.225 μg mL^-1^ for IHC [12, 28]. Mouse monoclonal antibodies against human LMα3 (clone CL3112) and mouse IgG (both Sigma-Aldrich, St. Louis, Missouri, USA) were used at 0.5 μg mL^-1^. Mouse monoclonal antibodies against GFP were used at 0.2 μg mL^-1^ for immunoblotting (clones 7.1 and 13.1, Sigma-Aldrich).

### Immunohistochemistry

Pilot tissues were obtained from the Liverpool Bio-Innovation Hub Biobank, all other TMA sections were purchased from Reveal Bioscience (product codes: BC02, BC03, BC05, BC06, and BC10; Reveal Bioscience, San Diego, USA) or US Biomax (product code: HBreD145Su02; US Biomax, Rockville, Maryland, USA). Sections were dewaxed and processed using a Leica Bond autostainer with Bond^™^ Polymer Refine Detection system (Leica Biosystems, Wetzlar, Germany). Briefly, following dewaxing, antigen retrieval was performed by incubating with a Tris/EDTA (pH 9 solution) solution for 20 min at 60°C, then endogenous peroxidases were blocked for 5 min at room temperature with Bond hydrogen peroxide solution. Sections were incubated with primary or isotype-matched control antibodies at room temperature for 30 min in Bond primary Ab solution (Tris-buffered saline, TBS, containing surfactant and protein stabiliser), then secondary anti-mouse IgG antibodies (<10 μg mL^-1^) with 10% v/v animal serum in TBS were added for 15 min at room temperature. DAB (66 mM) chromogen substrate was added for 20 min at room temperature, and counterstaining performed with 0.1% w/v haematoxylin for 5 min. At each stage, washing was performed with Bond wash solution (TBS containing surfactant). Sections were finally dehydrated through a series of ascending ethanol concentrations and then mounted with Pertex (all reagents Leica Biosystems). Stained tissue sections were imaged on the Aperio ImageScope slide scanner and processed using ImageScope software (Leica Biosystems).

### Immunohistochemistry interpretation

TMA cores were graded from 0–3 based on LaNt α31 immunoreactivity. Scores of 0 and 1 were then combined, and expression defined as low, medium, or high. All cores were scored by three independent scorers, and the mean score from duplicate cores used in final analyses. All patient data, including tumour/ node/ metastasis (TNM) status, tumour grade, and IHC marker scores (antigen ki-67 [Ki67], epidermal growth factor receptor [EGFR], human epidermal growth factor receptor 2 [Her2], oestrogen receptor [ER], and progesterone receptor [PR]) were provided by Reveal Biosciences. Data were rounded to the nearest integer for intensity scores where required. For Ki67 percentage cell staining, scores were grouped as 0, 6, 6–10, or >10%, as provided by Reveal Biosciences.

### Cell culture

MCF-7 [29] and MDA-MB-231 [30] cells were cultured in high glucose (4.5 g L^-1^) Dulbecco’s Modified Eagle Medium (DMEM) (Sigma-Aldrich) supplemented with 10% Foetal Calf Serum (FCS, LabTech International Ltd, Heathfield, East Sussex, UK) and 4 mM L-glutamine (Sigma-Aldrich).

### LaNt α31 expression

Full length *LAMA3LN1-eGFP* and *eGFP* adenoviral particles were prepared and used as previously described [12]. Transduction efficiency was determined by live fluorescent imaging at the time of analysis and expression confirmed by immunoblotting after 24 h. Cells were homogenised by scraping into urea/ sodium dodecyl sulphate (SDS) buffer (10 mM Tris-HCl pH 6.8, 6.7 M Urea, 1% w/v SDS, 10% v/v Glycerol and 7.4 μM bromophenol blue, containing 50 μM phenylmethysulfonyl fluoride and 50 μM N-methylmaleimide, all Sigma-Aldrich). Lysates were sonicated and 10% v/v β-mercaptoethanol added (Sigma-Aldrich). Proteins were separated by SDS-polyacrylamide gel electrophoresis (SDS-PAGE) using 10% polyacrylamide gels (1.5 M Tris, 0.4% w/v SDS, 10% acrylamide/ bis-acrylamide; electrophoresis buffer; 25 mM Tris, 190 mM glycine, 0.1% w/v SDS, pH 8.5 all Sigma-Aldrich). Proteins were transferred to nitrocellulose membranes (Biorad, California, USA) using the Biorad TurboBlot^™^ system and blocked for one hour at room temperature in Odyssey^®^ TBS-Blocking Buffer (Li-Cor BioSciences, Lincoln, Nebraska, USA). The blocked membranes were incubated overnight at 4°C with primary antibodies diluted in blocking buffer, then probed for 1 hour at room temperature with IRDye^®^ conjugated secondary antibodies against mouse IgG (800CW) raised in goat (LiCor BioSciences) diluted in Odyssey^®^ TBS-Blocking Buffer buffer at 0.05 μg mL^-1^. Membranes were imaged using the Odyssey^®^ CLX 9120 infrared imaging system and Image Studio Light v.5.2 (LiCor BioSciences) used to process scanned membranes.

### Proliferation assays

Proliferation assay: Cells were transduced with *LAMA3LN1-eGFP* or *eGFP* adenoviral particles. After 24 h, transduced or non-transduced cells were plated in triplicate at 2 x 10^3^ cells/well of a 96-well plate (Thermo Fisher Scientific, Waltham, Massachusetts, USA), with one group of non-transduced cells cultured in media containing Nocodazole at 20 ng/ mL-1 in culture media (Thermo Fisher Scientific). After 24 h Hoechst 33342 (Thermo Fisher Scientific) was added to the culture media, and cell nuclei imaged after ~20 min using the Cytivia IN Cell Analyzer 2500HS (BMG LABTECH, Marlborough, Massachusetts, USA). Representative proteins extracts were taken at the +24-hour timepoint. Cell nuclei were counted from 3 fields of view per well, for 3 separate wells per independent experimental repeat.

Proliferation inhibition assay: 1.25 x 10^5^ cells/well of non-transduced cells were seeded per well of a 6-well plate and treated with mitomycin c at 10 ug/ mL^-1^ for either 2 h or overnight. After 24 h, total RNA was extracted (Monarch^®^ Total RNA Miniprep Kit, NEB) and one-step RT-qPCR performed to assess LAMA3LN1 transcript abundance (Luna^®^ Universal qPCR Master Mix, NEB), using a Roche LightCycler^®^ 96 (Basel, Switzerland). GAPDH and RPLP0 were used as reference transcripts. All primer pairs were previously validated for efficiency and specificity [12], sequences as follows: LAMA3LN1: F-CTGGTGGAGGGGTCTGCATT, R-GGCAGTACACACAGGCTAAGAT; GAPDH: F-CGAGCCACATCGCTCAGACACC, R-GGTCAATGAAGGGGTCATTGATGGCAAC; RPLP0: F-GCAGCATCTACAACCCTGAAGTGCTTGA, R-GGTAGCCAATCTGCAGACAGACACTGG. The ΔCt method [31] was used to normalise the data to GAPDH and RPLP0, and the mean then compared to the untreated RNA (2^^-ΔΔCt^).

### 2D migration assays

For gap closure assays, cells were seeded into ibidi^®^ 2-well culture inserts (ibidi, Martinsried, Germany); at 7.0 x 10^4^ cells/well (MCF-7) or 8.0 x 10^4^ cells/well (MDA-MB-231). Culture inserts were carefully removed after 6 h, cell debris washed away, and the gap margin imaged using brightfield optics on a Nikon TiE epifluorescence microscope with a 10X objective at 0 and 16 h (Nikon, Tokyo, Japan). Gap closure was measured as a percentage relative to starting area using the freehand tool in image J (NIH, Bethesda, MA).

For low-density migration assays, cells were seeded at 5.0 x 10^4^ cells/well of a 12-well plate, then imaged every 2 minutes over a 2-hour period using a 20X objective on a Nikon Eclipse Ti-E fluorescent microscope adapted for live cell imaging. Individual cells were tracked using the MTrackJ plugin on image J and migration speed calculated.

### Inverted invasion assay

Inverted invasion assays were performed as previously described [32–34]. Briefly, 100 μL of 1 mg mL^-1^ rat tail collagen I (Corning Inc., New York, USA) or 4 mg mL^-1^ Matrigel^®^ from Engelbreth-Holm-Swarm (EHS) mouse sarcoma [35], was pipetted on top of Transwell^®^ 24-well, 0.8 μm, polycarbonate inserts (Corning). Then collagen was gelled through addition of 9.2 mM NaOH. Once gelled, the inserts were inverted and 100 μL of cell suspension containing 8.0 x 10^5^ cells were added to the lower surface. The transwells were then incubated for 4 h to allow the cells to attach before returning to the original position with basal side downward. 1 mL of serum-free media was pipetted into the lower chamber of the transwell, and 100 μL of normal culture media supplemented with 25 ng mL^-1^ EGF, as a chemoattractant, was added to the upper chamber [36]. After 72 h, the cells were fixed with 3.7% v/v formaldehyde for 30 min, permeabilised with 0.05% v/v Triton X-100 then stained for 1 hour with DAPI (Sigma-Aldrich). The inserts were mounted onto a glass coverslip and imaged with a Zeiss Marianas (3i) spinning-disk confocal microscope by taking a z-stack with images every 5 μm, using SlideBook 5.5 software (3i, Intelligent Imaging Innovations Ltd, London, UK).

An algorithm was generated to automatically measure the DAPI stained nuclei in each slice of the z-stack. The average nuclei size was established by taking a range of manual measurements through the different planes of the z-stack. The average was used to set: i) the intensity threshold for distinguishing nuclei fluorescence from background as *t = 1000*, ii) the expected nuclei size *s = 240* pixels, corresponding to an area of 101.4 μm or a radius of 5.68 μm, ii) the lower size threshold *s^- = 120* pixels (area = 50.7 μm or a = radius of 4.02 μm), below which captured objects were not considered to be nuclei and iv) the upper size threshold *s^+ = 400* pixels (area 169 μm or radius of 7.33 μm), above which the captured object was assumed to be an artefact. For each image slice, the image was imported and a coarse segmentation of the nuclei performed by thresholding at the intensity value *t* to achieve a binary nuclei/non-nuclei image. Any nuclei within an absolute distance of 0.65 μm or 1 pixel were connected to take into account noise, and an initial index taken of the distinct identified nuclei candidates, while measuring the size of each nuclei.

Individual cells were then identified as follows: i) any identified object under *s^-*, *s/2* pixels or over *s^+* pixels in size was considered to be noise, ii) any captured object within the thresholds *s/2* and *3s/2* was considered to be one nuclei, iii) any remaining captured object which was larger than *3s/2* in size was considered to be a cluster of nuclei which could not be split due to the resolution of the image. In these cases, the number of cells in the cluster was calculated as *(cluster size) / s*, rounded to the nearest integer. The following measures were then taken: i) total luminance of the cell image, ii) PixCount: the number of pixels considered to be a cell after thresholding, corresponding to the total area in microns of the region considered to contain a cell. iii) Cell Count: the total number of cells identified, including those estimated from cell clusters, iv) Entropy: a measure of randomness of the thresholding data, which identified how clustered the cells in the image were.

### Data analyses

Microsoft Excel (Microsoft, Washington. USA), Graphpad Prism v.6 (Graphpad Software, California, USA), SPSS statistic 24 (IBM Corporation, New York, USA), or Jupyter Notebook (python) were used to analyse numerical data and generate graphs. Figures were generated using CoralDraw 2017 or Adobe Illustrator. The Wilcoxon signed-rank test or Somers’ D was used for ordinal data, or Mantel-Cox log-rank test for survival data in immunohistochemistry analyses, and one-way rANOVA or ANOVA with Bonferroni post hoc test were used for continuous variables in gap closure, single cell migration, cell invasion depth, and proliferation data, or 2-way ANOVA with Tukey’s post hoc test for entropy. Differences were deemed statistically significant where type I error rates were below 5%.

## Results

### LaNt α31 and LMα3 display distinct distribution patterns in invasive ductal carcinomas

**Fig 1:**
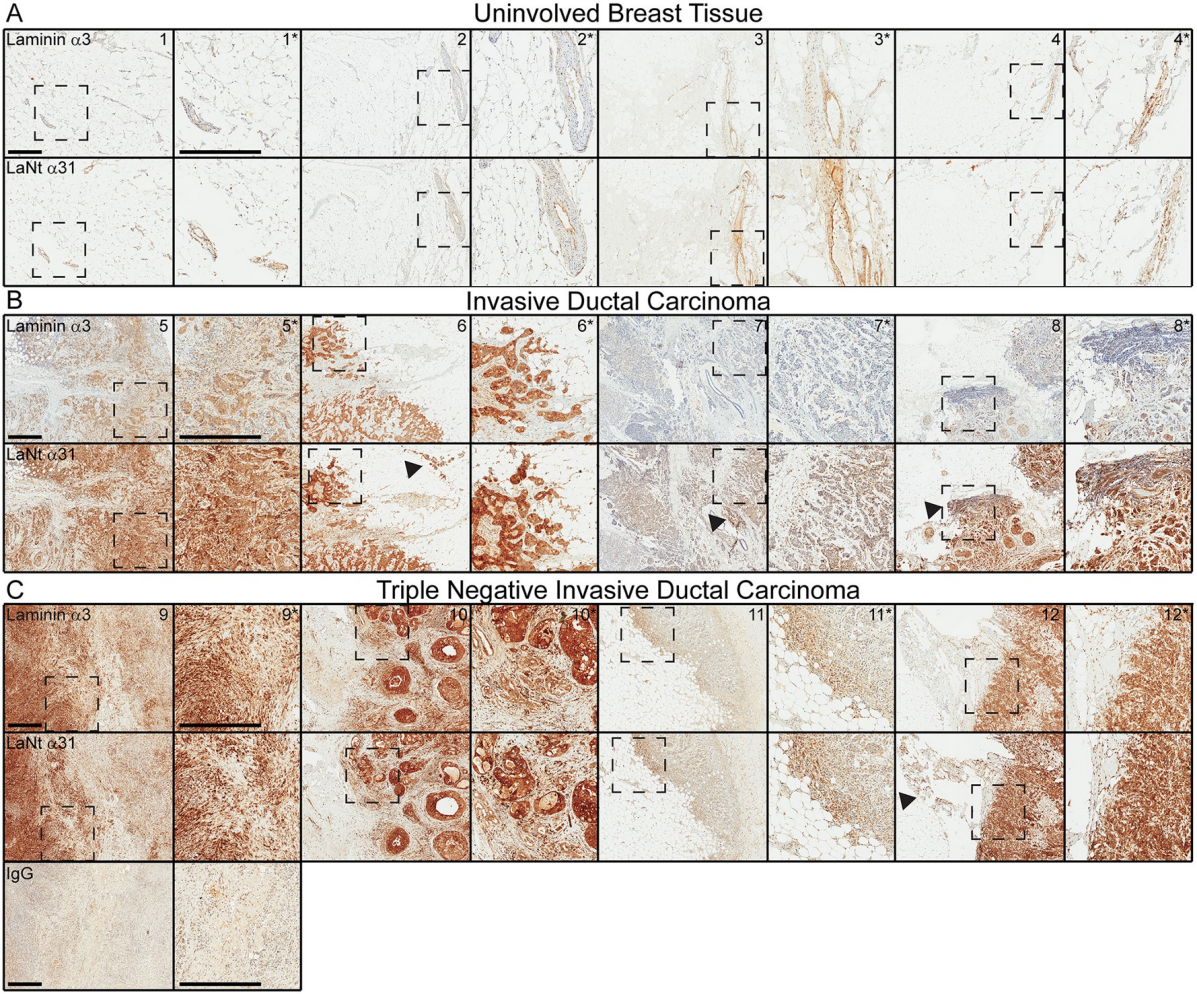
LaNt α31 is upregulated in invasive ductal carcinoma. Serial sections from formalin-fixed paraffin-embedded human breast tissue processed for immunohistochemistry with mouse monoclonal antibodies against laminin α3, LaNt α31, or mouse IgG- isotype control, (A) uninvolved breast tissue, (B) invasive ductal carcinoma, and (C) ER-, PR-, Her- invasive ductal carcinoma. Dashed boxed regions are shown at higher magnification in columns to the right. Arrowheads indicate regions of anti-LaNt α31 immunoreactivity not recognised by anti-laminin α3. Scale bars: 500 μm.

First we compared the distribution of LaNt α31 and LMα3 in a pilot panel of four normal (Fig 1A), four invasive ductal carcinoma (Fig 1B) and four invasive ductal carcinoma that were triple negative for ER, PR, and Her2 (Fig 1C). The LMα3 antibodies used recognise both the LMα3a and LMα3b forms. LaNt α31 and LMα3 displayed very similar distribution in the normal tissue (Fig 1A), primarily restricted to TDLU as previously reported [28], with the most intense immunoreactivity being present in the extracellular matrix surrounding the TDLU. However, much stronger and more widespread cellular LaNt α31 expression was detected in three of the four invasive ductal carcinoma and three of the four triple negative specimens. In the invasive ductal tissue, LaNt α31 displayed a more widespread distribution than LMα3 (arrowheads, Fig 1B), whereas in ER-PR-Her2- cases the LMα3 and LaNt α31 distribution were very similar (Fig 1C). LMα3 has been extensively investigated in breast cancer [23–25]; however, these new data for LaNt α31 indicated potential additional value of investigating this isoform independently from LMα3 in invasive ductal carcinoma.

### LaNt α31 expression is elevated in invasive ductal carcinoma and in nodal metastases compared to primary tumour tissue

**Fig 2:**
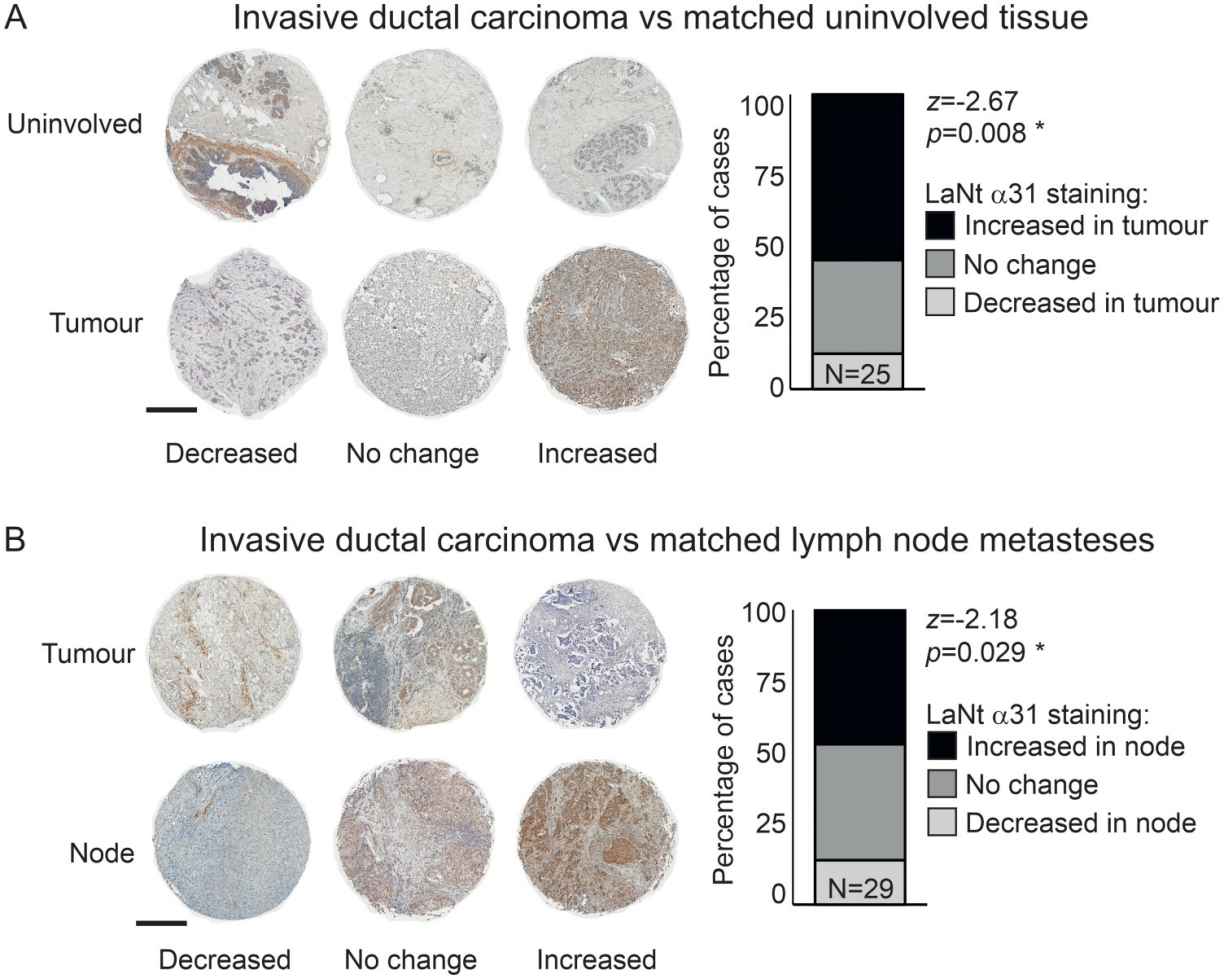
LaNt α31 is upregulated in ductal carcinoma and in lymph node metastases. Formalin-fixed paraffin-embedded human breast tissue microarray sections processed for immunohistochemistry with mouse monoclonal antibodies against LaNt α31. Two separate arrays were used; (A) uninvolved with paired invasive/ in situ ductal carcinoma tissues (N = 25), (B) invasive ductal carcinoma with paired node metastases (N = 29). Cores were scored as either decreased, no change, or increased staining intensity relative to the paired uninvolved (A) or primary tumour (B) core from the same donor (representative images shown). Stacked columns of percentage of cases in each category were plotted and Wilcoxon signed ranks test used to describe observed relationship. Scale bars: 500 μm.

To formally determine whether LaNt α31 expression levels change in invasive ductal carcinoma, the relative intensity of cellular immunoreactivity in epithelial-like tissue was compared between normal breast tissue and tumour biopsies from the same person, with intensity scored by three independent, blinded scorers (representative examples, Fig 2A, all cores S1A Fig, patient ages Table 1). These paired analyses revealed LaNt α31 expression to be increased in the cancer specimen in 14 of 25 tumours (56%), eight had no change, while three displayed decreased expression in the cancer tissue (Wilcoxon signed ranks test, z = -2.67, p = 0.008, Fig 2A).

**Table 1:** Patient ages for each study cohort.

Study cohort	Mean	Median	Range	N
Invasive ductal carcinoma vs uninvolved^1^	46	45	29–70	25
Invasive ductal carcinoma vs lymph node metastases^2^	47	46	30–69	29
Invasive ductal carcinoma grade, nodal involvement, biomarker correlative analyses^3^	47	44	27–86	198*
Invasive ductal carcinoma survival analyses^4^	60	57	33–88	126

^1^Fig 1,

^2^Fig 2,

^3^Figs 3 and 4,

^4^Fig 3E.

*full data not available for all specimens

Comparison of staining intensity between cores taken from the primary tumour against those from nodal metastasis from the same person using the same scoring approach, revealed that 13 of the 29 (45%) of the nodal metastasis displayed stronger LaNt α31 staining compared with primary tumour tissue, 12 were scored the same, and four were decreased in the nodal tissue (z = -2.18, p = 0.029, Fig 2B, S1B Fig, patient ages Table 1).

### High LaNt α31 expression is associated with more proliferative tumours

**Fig 3:**
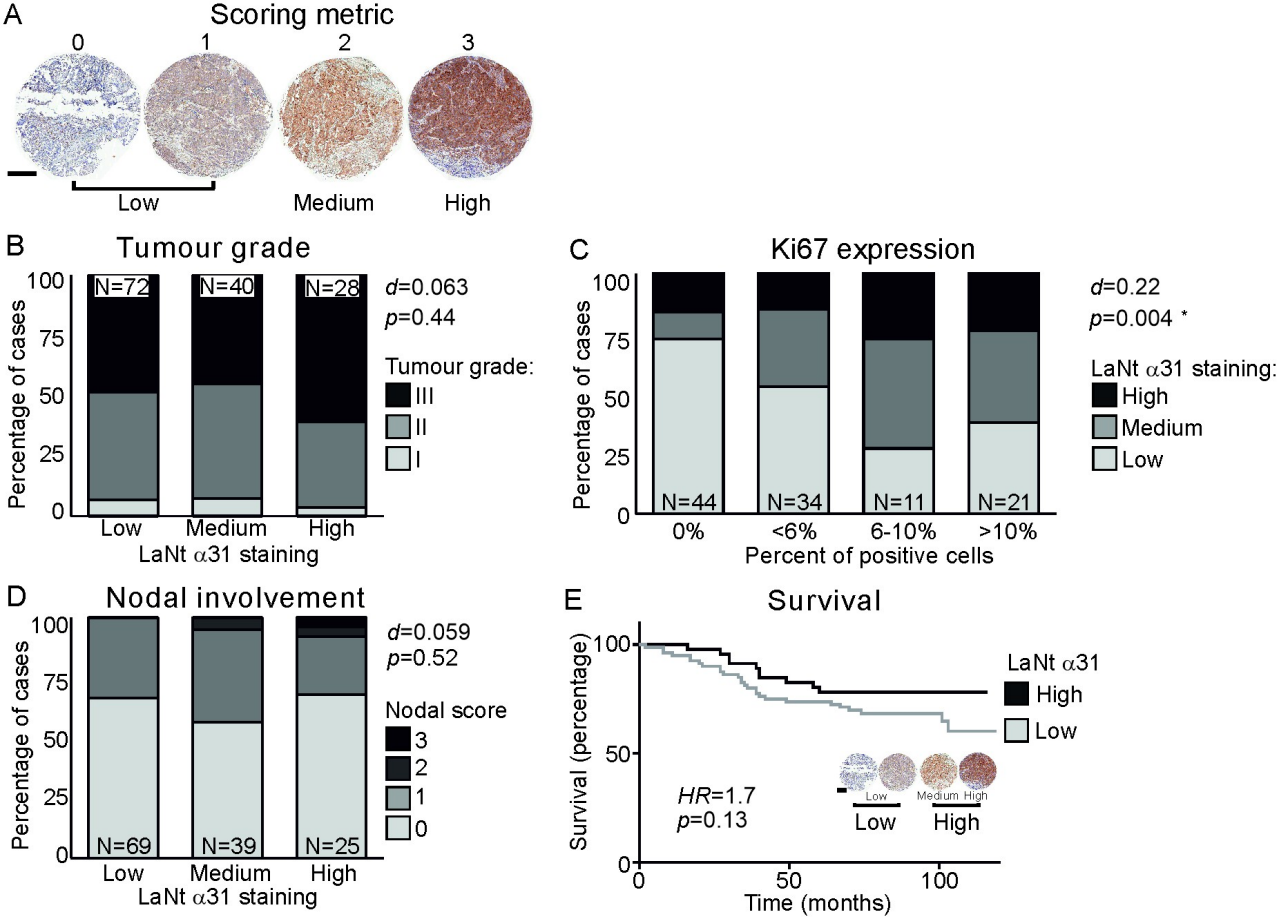
LaNt α31 upregulation in invasive ductal carcinoma does not correlate with nodal involvement or tumour grade. Formalin-fixed paraffin embedded human breast tissue microarray sections processed for immunohistochemistry with mouse monoclonal antibodies against LaNt α31. Cores were scored based on LaNt α31 staining intensity from 0–3. Scores of 0 or 1 were combined and designated as low LaNt α31 expression, score 2 as medium expression, and 3 as high expression. (A) Representative example of core scoring. (B-D) Stacked column graphs of percentage of cases with each staining intensity segregated by tumour grade I, II, or III (B), Ki67 expression (C), or by nodal involvement (D). Somers’ D was used to describe observed relationships between LaNt α31 staining intensity and the independent variables. (E) Kaplan–Meier survival curve, where LaNt α31 staining intensity was simplified to low or high by pooling medium and high cores. Logrank test was used to determine hazard ratio and chi square for significance. Scale bar in (A): 300 μm.

To determine if LaNt α31 immunoreactivity held prognostic value for invasive ductal carcinoma, we processed cores from 324 patients (Table 1, two cores per patient) and scored them by three independent scorers as “low”, “medium” or “high” LaNt α31 intensity (representative images Fig 3A). First, we asked if LaNt α31 staining intensity was predictive for tumour grade, Ki67 expression (as a marker of proliferation [37, 38]), nodal involvement, or survival (Fig 3B–3E). No association was observed between LaNt α31 staining intensity and tumour grade (Somers’ d = 0.063, p = 0.44, Fig 3B); however, there was a positive correlation between Ki67 expression level and LaNt α31 intensity (d = 0.22, p = 0.004 Fig 3C). The relative proportion of high, medium and low LaNt α31 did not correlate with nodal involvement (d = 0.059, p = 0.52, Fig 3D). Stratifying the cohort based on Ki67 expression levels before analysing association with nodal involvement, did not change this lack of association.

Survival was assessed for 126 cases where these data were available. To account for the smaller sample size, LaNt α31 staining intensity was simplified to either low or high expression by combining the medium with the high expression cases. There were no statistically significant differences in survival between high and low expression groups (low = 66% survival, high = 78% survival, hazard ratio 1.75, confidence interval 0.9–3.4, p = 0.13, Fig 3E).

**Fig 4:**
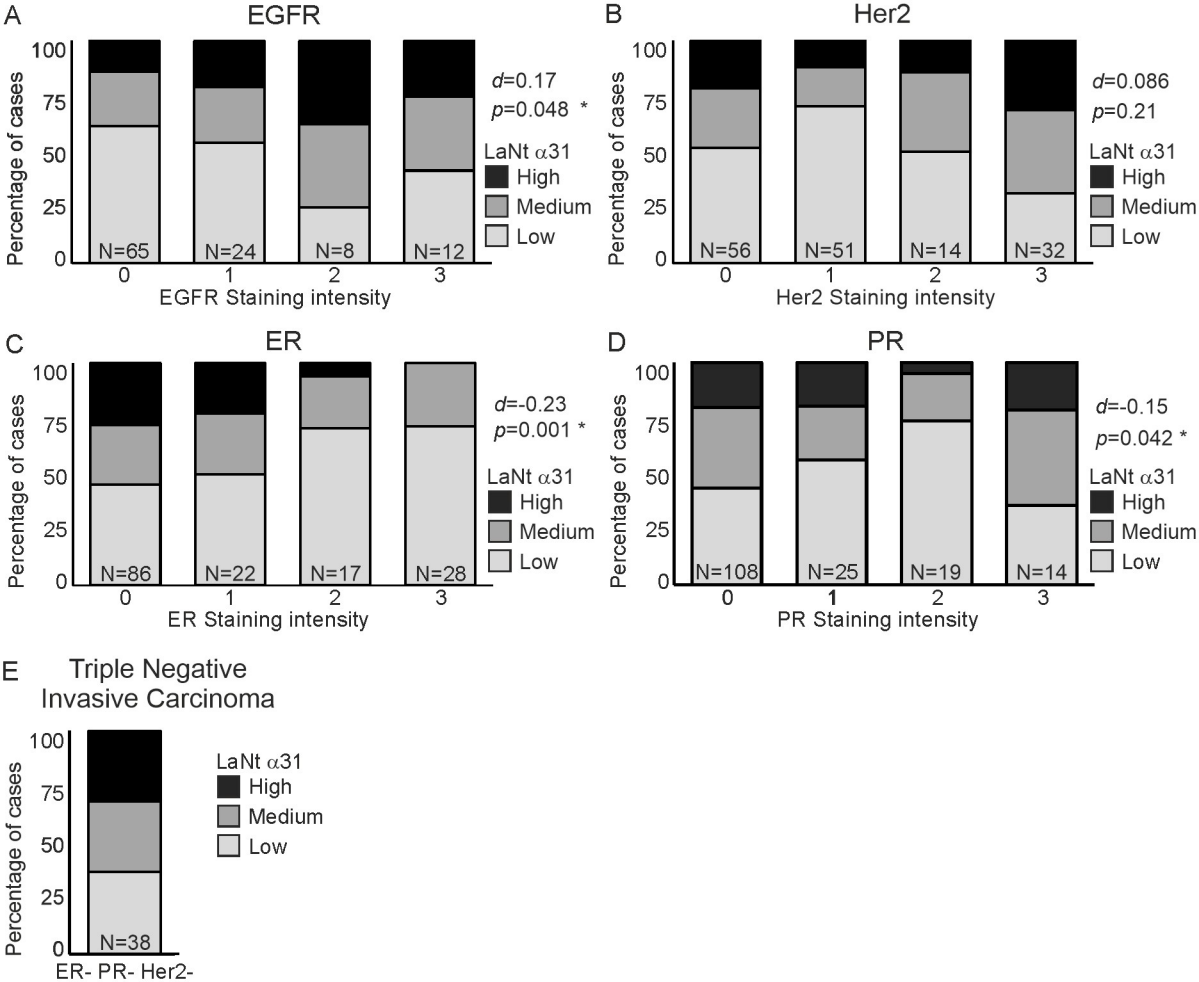
Positive correlation between LaNt α31 staining intensity and EGFR expression. Formalin-fixed paraffin embedded human breast tissue microarray sections processed for immunohistochemistry with mouse monoclonal antibodies against LaNt α31. Cores were scored based on LaNt α31 staining intensity from 0–3. Scores of 0 or 1 were combined and designated as low LaNt α31 expression (light grey bars), scores 2 as medium expression (dark grey bars), and 3 as high expression (black bars). Stacked column graph of percentage of cases that fall into group after segregation based on pathologist provided grading of immunohistochemistry markers; (A) EGFR, (B) Her2, (C) ER, (D) PR, or (E) ER- PR- Her2- cases. Somers’ D was used to describe observed relationship between LaNt α31 staining intensity and independent variable.

Next, we asked if LaNt α31 staining displayed a relationship with any of the commonly used breast cancer biomarkers (Fig 4). These data revealed a weak but statistically significant positive association between LaNt α31 and EGFR, and a weak but statistically significant negative association between ER and PR (EGFR d = 0.17, p = 0.048, ER d = -0.23, p = 0.001; PR d = -0.15, p = 0.042, Fig 4A) but no association with Her2 (Her2 d = 0.086, p = 0.21, Fig 4B).

The cores from triple negative cancers were analysed separately. These data revealed that 63% of the ER- PR- Her2- cores had either medium or high LaNt α31 staining intensity (Fig 4E, n = 38 cores).

### Induced LaNt α31 expression reduces breast cancer cell proliferation

Although the immunohistochemistry findings indicated association between higher LaNt α31 staining and higher Ki67 expression, this does not necessarily imply a causative relationship nor does it indicate directionality.

To investigate if LaNt α31 expression is linked to proliferation, we used inhibited cell division using mitomycin c in two widely used cell lines derived from malignant ductal carcinomas, MCF7 and MDA-MB-231, then quantified *LAMA3LN1* transcript abundance using RT-qPCR normalised to GAPDH and RPLP0. Analysis revealed that cell proliferation is not required for LaNt α31 transcript expression in either MCF7 (mean fold change relative to untreated cells ± S.D: +2 h mitomycin c 1.2 ± 0.29, +16 h mitomycin c 0.73 ± 0.12 n≥ 4. rANOVA on mean ΔCt values, *p* = 0.016, not significant when adjusted for multiple comparisons, S2 Fig) or MDA-MB-231 cells (+2 h mitomycin c 1.1 ± 0.19, MDA-MB-231 +16 h mitomycin c 0.89 ± 0.095, n≥ 4. rANOVA on mean ΔCt values, *p* = 0.066, S2 Fig).

**Fig 5:**
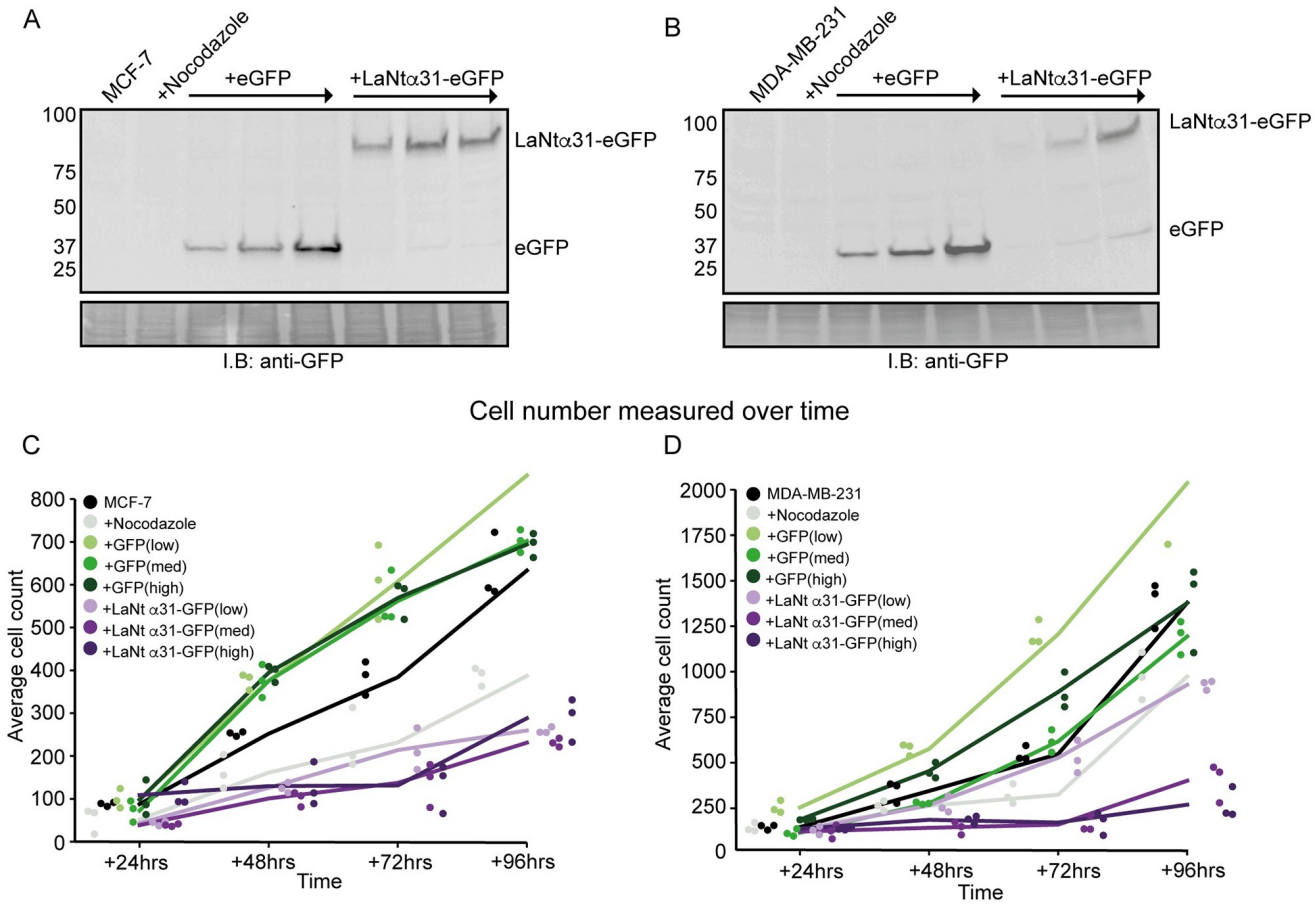
LaNt α31 overexpression reduces proliferation rates. MCF-7 or MDA-MB-231 cells, either left untreated, treated with 20 ng mL-1 nocodazole, or transduced with increasing doses of eGFP (+eGFP) or LAMA3LN1-eGFP (+LaNt α31-eGFP) adenoviral particles, were cultured for 96 h following transduction and replating. (A) Immunoblots from total cell lysates for MCF-7 or MDA-MB-231 cells taken after 24 h were probed with antibodies against eGFP, with ponceau S total protein-stained membrane shown below. (B) Hoechst 33342 was added to the culture media, and the cell nuclei imaged after 20 min. Each dot represents an experimental repeat consisting of the mean of 3 fields of view per well for 3 technical replicates.

Next, to determine whether LaNt α31 upregulation influences cell proliferation, we used an adenoviral system to drive overexpression of LaNt α31 tagged with eGFP (+LaNt α31-eGFP) in MCF-7 and MDA-MB-231 (Fig 5A and 5B). Cells transduced with eGFP only were used to control for adenoviral transduction and eGFP expression. Viral load was functionally titred to achieve low, medium and high levels of expression (Fig 5A and 5B). Expression of LaNt α31-eGFP caused a reduction in cell numbers in MCF-7 at all expression levels (Fig 5C and 5D, mean nuclei count at +96 h ± S.D: untreated MCF-7 633 ± 80, nocodazole 387 ± 70, +eGFP low 855 ± 90, +eGFP med 702 ± 60, +eGFP high 694 ± 30, +LaNt α31-eGFP low 260 ± 10, +LaNt α31-eGFP med 231 ± 10, +LaNt α31-eGFP high 288 ± 50. rANOVA controlling for multiple comparisons, *p* = 0.004). A similar reduction in cell numbers was also identified in MDA-MB-231 cells (untreated MDA-MB-231 1379 ± 80, +nocodazole 974 ± 20, +eGFP low 2037 ± 90, +eGFP med 1194 ± 30, +eGFP high 1378 ± 30, +LaNt α31-eGFP low 928 ± 10, +LaNt α31-eGFP med 401 ± 10, +LaNt α31-eGFP high 270 ± 50. rANOVA controlling for multiple comparisons, *p* = 0.006).

### LaNt α31 expression does not induce increased cell migration rates in low-migratory breast cancer cells

**Fig 6:**
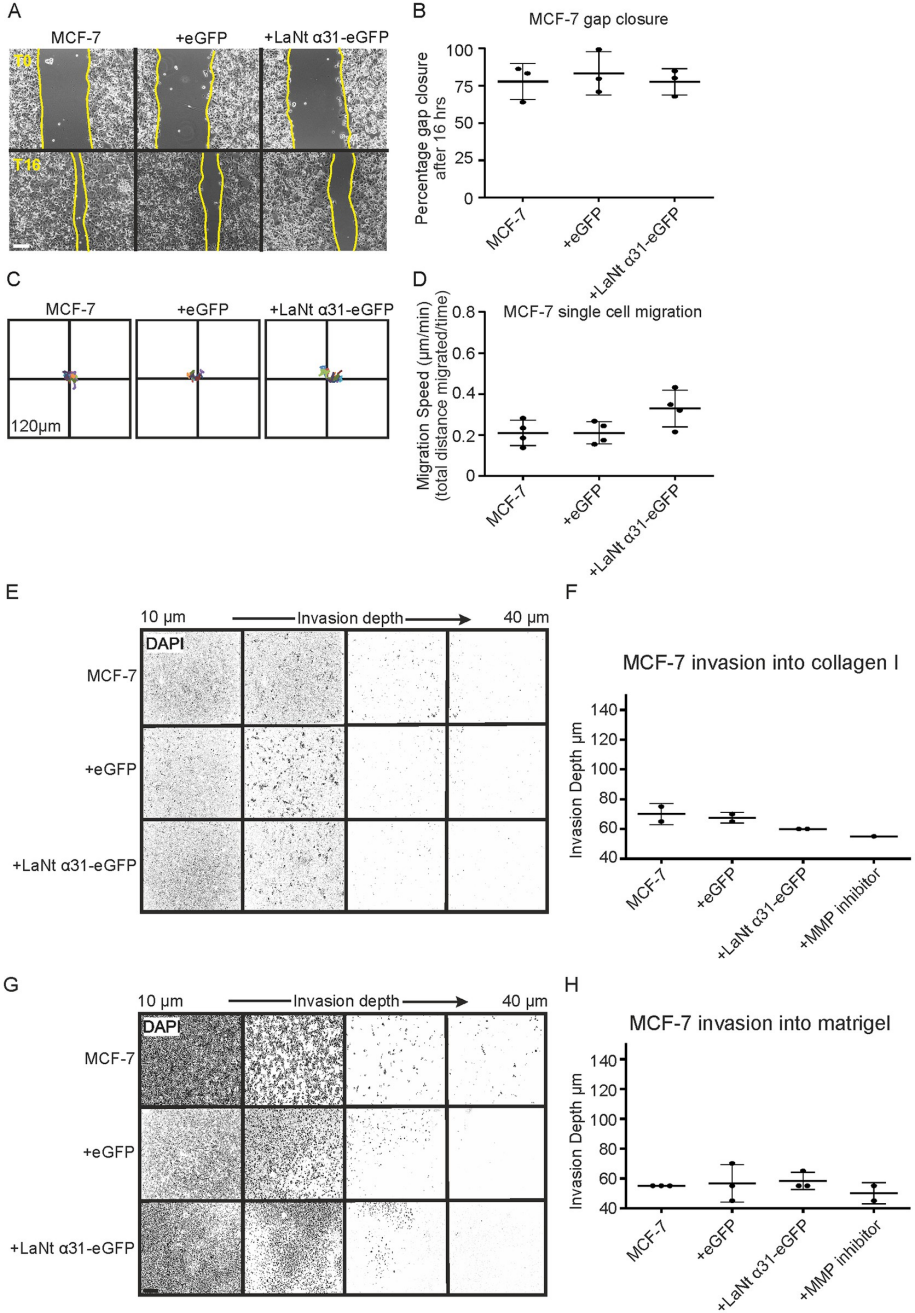
LaNt α31 overexpression does not significantly affect 2D migration or 3D invasion of MCF-7 cells. MCF-7 cells were either left untreated or transduced with eGFP (+eGFP), or LAMA3LN1-eGFP (+LaNt α31-eGFP). For gap closure assays, 24 h after transduction, cells were seeded into ibidi^®^ 2-well culture inserts and allowed to attach for 6 h, the inserts were then removed, and the gap margin imaged at 0 h and 16 h. For single cell migration assays, 24 h after transduction, cells were seeded onto tissue culture plastic and the migration paths of individual cells tracked over a four-hour period. (A) Representative images from immediately after removing chamber (T0 upper panels) and after 16 h (T16 lower panels), yellow lines delineate wound margins. (B) Gap closure was measured as a percentage relative to starting gap area. (C) Vector diagrams showing representative migration paths of 10 individual cells with each colour representing a single cell. (D) Migration speed was measured as total distance migrated over time. Each point on the associated dot plots represents an independent experiment with 2–3 technical replicates per experiment for gap closures assays or 20–40 cells per low density migration assay. For invasion assays, cells were plated onto the outside of a transwell membrane. 10 ng mL^-1^ epidermal growth factor was used to stimulate invasion through the membrane and into collagen I or Matrigel. After 48 h, the cells were fixed and stained with DAPI then imaged at 5 μm intervals using a spinning disk confocal microscope. (E and G) Representative images of invasion into collagen I or Matrigel from 10–40 μm presented at equal intervals. (F and H) Absolute invasion depth was measured where cell count ≥1. Treatment with GM6001 MMP inhibitor was included as an invasion inhibiting control. Each point on the graphs represents an independent experiment, with 2–3 technical replicates per assay. Statistical tests of differences relative to controls were performed using one-way rANOVA followed by Bonferroni’s post hoc analyses; p>0.05 in all comparisons. Scale bar in (a) represents 100 μm.

MCF-7 cells inherently migrate slowly [39–41]. This line was therefore used to assess if induction of LaNt α31 expression increases migration. In gap closure assays (Fig 6A and 6B) and single cell migration assays (Fig 6C and 6D), no statistically significant differences were observed between MCF-7 cells expressing LaNt α31 and controls (Gap closure: median MCF-7 83.3%, +eGFP 79.7%, +LaNt α31-eGFP 80.2%, *p* = 0.38, Single cell assays: mean migration speed ± S.D. MCF-7 0.21 μm min^-1^ ± 0.06, +eGFP 0.21 μm min^-1^ ± 0.05, +LaNt α31-eGFP 0.33 μm min^-1^ ± 0.09, *p* = 0.53, determined by rANOVA).

MCF7 cells also do not display inherent invasive behaviour [39–41]. We, therefore, next asked if increasing LaNt α31 expression could induce invasive capabilities in MCF7 by using an inverted invasion assay [34, 42], where cells were seeded on the base of a porous membrane then stimulated to invade into a provided matrix and using an EGF gradient as a chemoattractant (Fig 6E–6H). As we hypothesised that LaNt α31 effects could be LM specific, invasion into two different matrices were analysed; collagen I to mimic the interstitial matrix (Fig 6E and 6F) and Matrigel, a BM analogue that contains approximately 60% LM111, 30% Type IV collagen, and 8% entactin [35, 43] (Fig 6G and 6H). Untreated MCF-7 cells and cells transduced with either GFP or LaNt a31-GFP each had very low invasive capabilities into either matrix type with no significant change in invasion depth between the different conditions (mean ± S.D invasion depth into collagen I: MCF-7 70 μm ± 7, +eGFP 67 μm ± 4, +LaNt α31-eGFP 60 μm ± 0, *p* = 0.22, determined by ANOVA, Fig 6F. Into Matrigel: MCF-7 55 μm ± 0, +eGFP 57 μm ± 13, +LaNt α31-eGFP 58 μm ± 6, *p* = 0.71, determined by rANOVA, Fig 6H).

### Increased LaNt α31 expression causes a change in mode of invasion into laminin-rich hydrogels

**Fig 7:**
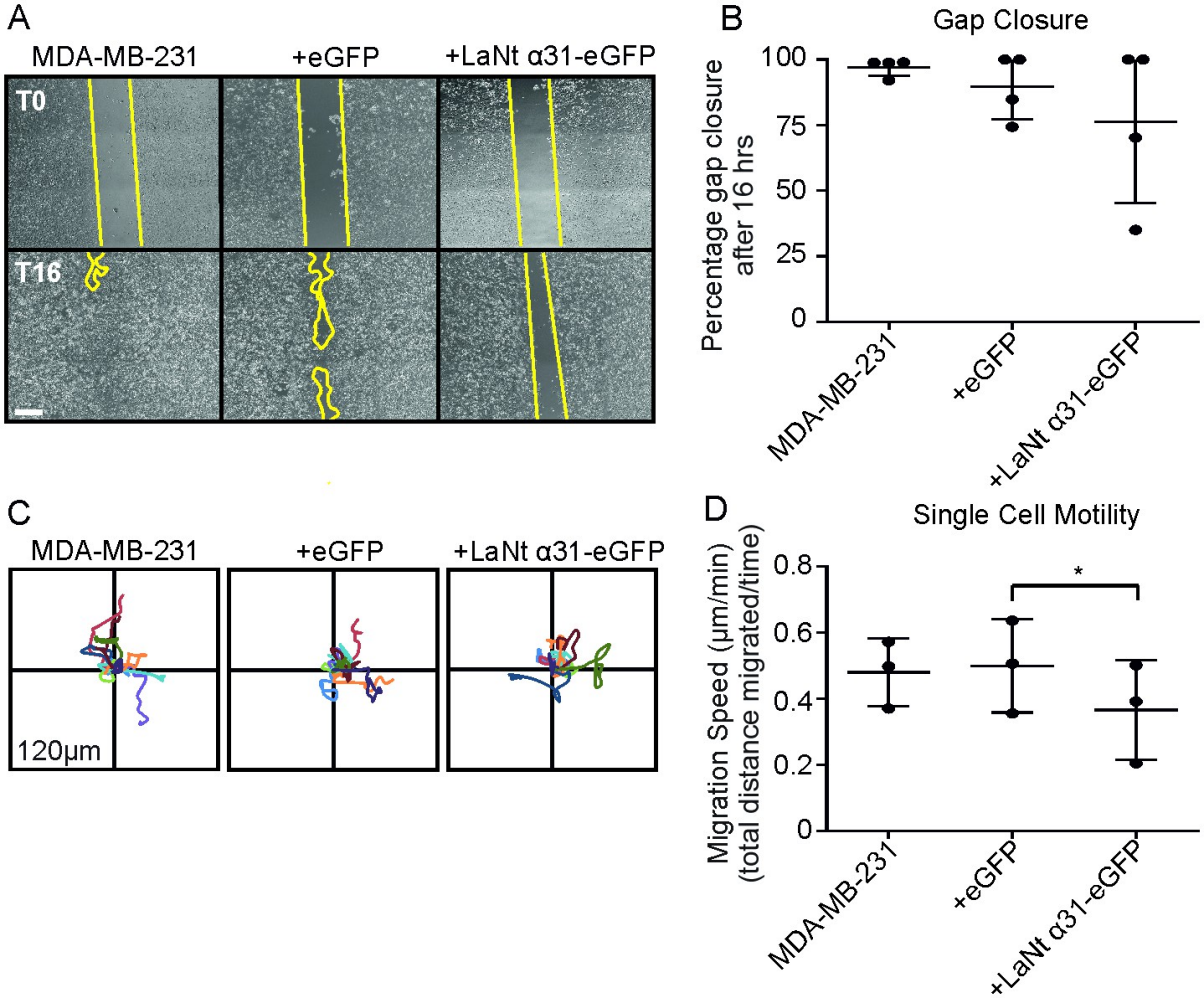
LaNt α31 overexpression does not significantly affect 2D migration MDA-MB-231 cells. MDA-MB-231 cells were either left untreated or transduced with eGFP (+eGFP), or LAMA3LN1-eGFP (+LaNt α31-eGFP). For gap closure assays, 24 h after transduction, cells were seeded into ibidi^®^ 2-well culture inserts and allowed to attach for 6 h, the inserts were then removed, and the gap margin imaged at 0 h and 16 h. For single cell migration assays, 24 h after transduction, cells were seeded onto tissue culture plastic and the migration paths of individual cells tracked over a four-hour period. (A) Representative images from immediately after removing chamber (T0 upper panels) and after 16 h (T16 lower panels), yellow lines delineate wound margins. (B) Gap closure was measured as a percentage relative to starting gap area. (C) Vector diagrams showing representative migration paths of 10 individual cells with each colour representing a single cell. (D) Migration speed was measured as total distance migrated over time. Each point on the associated dot plots represents an independent experiment with 2–3 technical replicates per experiment for gap closures assays or 20–40 cells per low density migration assay. Statistical tests of differences relative to controls were performed using one-way rANOVA followed by Bonferroni’s post hoc tests; * p<0.05. Scale bar in (a) represents 100 μm.

In contrast to MCF7, MDA-MB-231 are much more motile and invasive [34, 42, 44]. The effects of induced LaNt α31 expression on gap closure (Fig 7A and 7B) and single cell migration (Fig 7C and 7D) of MDA-MB-231 were below the threshold for statistical significance with one exception between eGFP and +LaNt α31-GFP in single cell migration assays (Gap closure; median: MDA-MB-231 98.8%, +eGFP 92.7%, +LaNt α31-eGFP 85.1%, *p* = 0.23, low density migration, mean speed ± S.D: MDA-MB-231 0.48 μm min^-1^ ± 0.10, +eGFP 0.50 μm min^-1^ ± 0.2, +LaNt α31-eGFP 0.37 μm min^-1^ ± 0.2, MDA-MB-231 vs +LaNt α31-eGFP, *p* = 0.16; +eFGP vs +LaNt α31-eGFP, *p* = 0.02 determined by rANOVA with Bonferroni post hoc test.).

**Fig 8:**
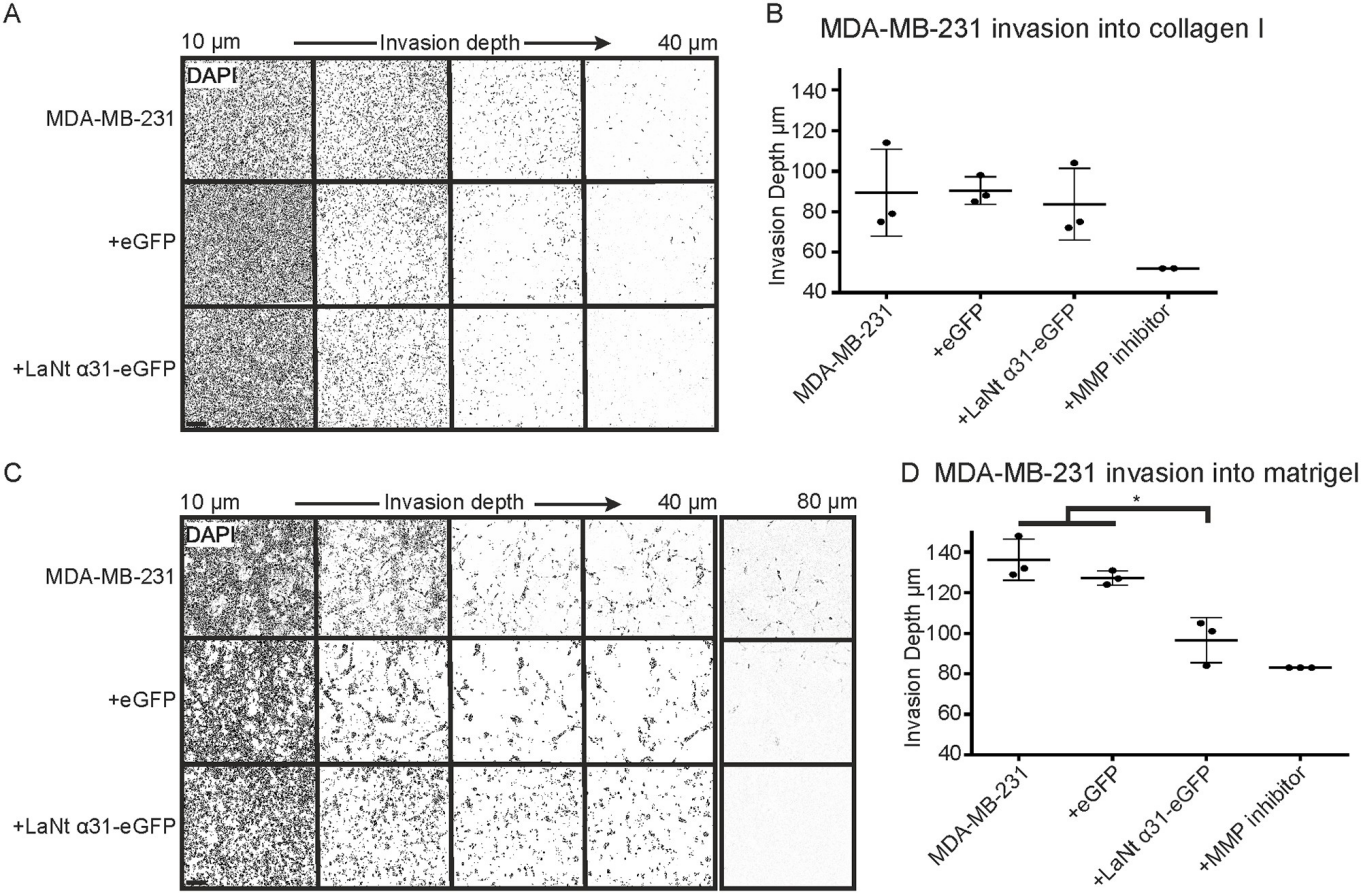
LaNt α31 overexpression causes a small reduction in invasion of MDA-MB-231 cells into Matrigel. MDA-MB-231 cells, either left untreated or transduced with eGFP (+eGFP), or LAMA3LN1-eGFP (+LaNt α31-eGFP), were plated onto the outside of a transwell membrane. 10 ng mL^-1^ epidermal growth factor was used to stimulate invasion through the membrane into collagen I (A-B) or Matrigel (C-D). After 48 h, the cells were fixed and DAPI stained then imaged at 5 μm intervals using a spinning disk confocal microscope. (A) and (C) Representative images from 10–40 μm depth presented at equal intervals, with an additional slice at 80 μm in (C). Absolute invasion depth was measured where cell count ≥1. Treatment with GM6001 MMP inhibitor was included as an invasion inhibiting control. Each point on the graphs in (B) and (D) represents an independent experiment, with 2–3 technical replicates per assay. * represents p<0.05 between bracketed groups as determined by one-way ANOVA followed by Bonferroni’s post hoc analyses.

As expected, MDA-MB-231 invaded efficiently into collagen I and Matrigel matrices (Fig 8). The invasion depth of +LaNt α31-eGFP MDA-MB-231 cells into collagen I was unchanged compared with controls (mean ± S.D depth into collagen: MDA-MB-231 89 μm ± 21, +eGFP 90 μm ± 7, +LaNt α31-eGFP 83 μm ± 18, *p* = 0.52 rANOVA, Fig 8A and 8B). However, a small reduction was observed in the total invasion depth into Matrigel in the LaNt α31 induced expression cells relative to controls (invasion into Matrigel: MDA-MB-231 136 μm ± 10 S.D, +eGFP 127 μm ± 4, +LaNt α31-eGFP 97 μm ± 11, MDA-MB-231 vs +LaNt α31-eGFP *p* = 0.005, +eGFP vs +LaNt α31-eGFP *p* = 0.017, rANOVA with Bonferroni post hoc test. Fig 8C and 8D).

**Fig 9:**
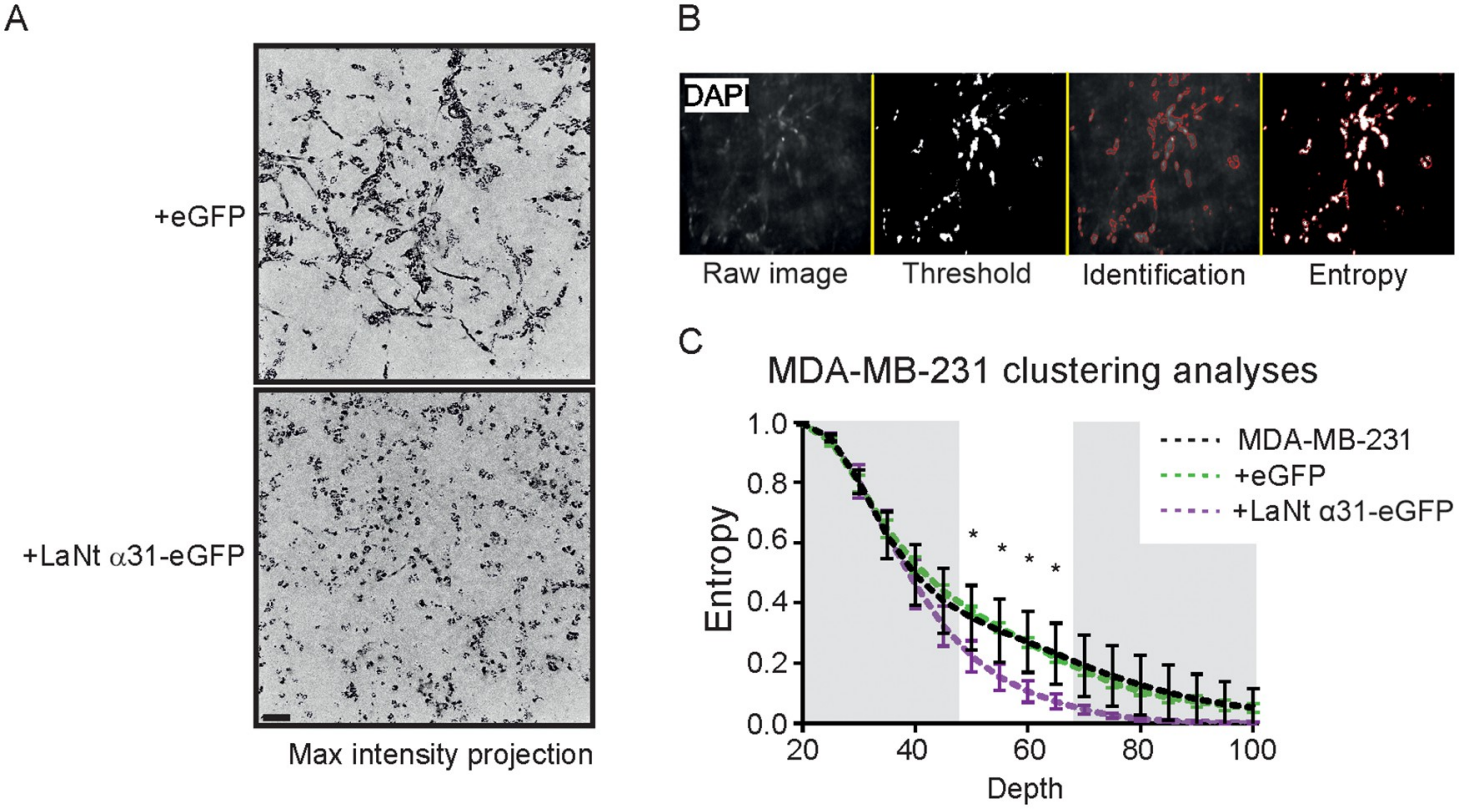
LaNt α31 overexpression causes a change in mode of invasion of MDA-MB-231 cells into Matrigel. (A) Maximum intensity projection of planes from 20–60 μm from the same assays in Fig 8C. (B) Image analyses method for determining entropy as a measure of cell clustering/cohesiveness; each stack of images was processed using an automated processing algorithm, where cell count and entropy score after a threshold was measured for each image in the stack. (C) Entropy score versus depth graph with points representing mean and SD from 3 independent experiments. Shaded regions indicate where comparisons lack value due to either high cell counts (0–45 μm) or differences in cell numbers between conditions (>70 μm). * denote statistically significant differences between +LaNt α31-GFP cells and both MDA-MB-231 and +GFP conditions by 2-way ANOVA with Tukey’s post hoc test.

Although the differences in invasion depth were relatively small, visual analysis of the Matrigel invasion assays revealed a clear and intriguing distinct phenotypic difference between the LaNt α31 overexpressing cells compared with the controls (Figs 8C and 9A). Whereas MDA-MB-231 cells usually invade into Matrigel as multicellular streams, as has been reported previously [44, 45], the +LaNt α31-eGFP cells did not display this behaviour (S1 and S2 Movies, maximum intensity projection Fig 9A). To assess this quantitatively, we wrote a macro to convert the DAPI stained images into a measure of cohesiveness at each depth (entropy) (Fig 9B). Between 50 and 65 μm depth, cell densities were similar between +LaNt α31-eGFP and controls, therefore allowing direct comparison between lines. Across this depth range the level of cohesiveness was statistically significantly lower in the +LaNt α31 MDA-MB-231 cells than in both control treatments (Mean+SD entropy MDA-MB-231 / +eGFP / +LaNt α31-eGFP: 50 μm, 0.35 ± 0.09, 0.37 ± 0.01, 0.22 ± 0.04; 55 μm, 0.31 ± 0.09, 0.32 ± 0.01, 0.15 ± 0.04; 60 μm 0.27 ± 0.08, 0.27 ± 0.01, 0.11 ± 0.03; 65 μm 0.23 ± 0.08, 0.22 ± 0.01, 0.07 ± 0.02, p<0.05 compared for +LaNt α31-eGFP compared with MDA-MB-231 and +eGFP for all depths between 50 and 65 μm, 2-way ANOVA Tukey’s post hoc test, Fig 9C).

### Tumours with high LaNt α31 expression are likely to be non-cohesive

**Fig 10:**
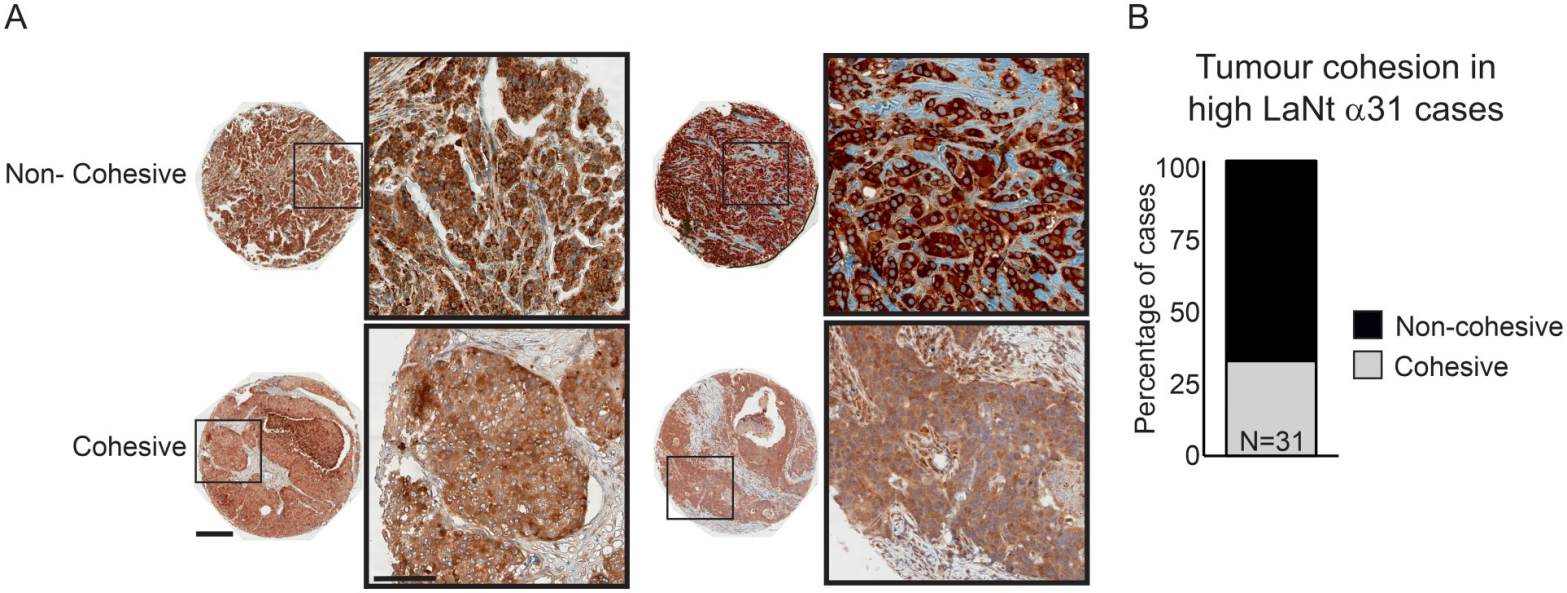
High LaNt α31 expression is associated with low tumour cohesion in invasive ductal carcinoma. Formalin-fixed paraffin embedded human breast tissue microarray sections processed for immunohistochemistry with mouse monoclonal antibodies against LaNt α31. Tumour cohesion was graded as either cohesive (tight tumour islands), or non-cohesive (chord-like) in tumour cores scored as having high LaNt α31 expression. (A) Representative example of core grading. (B) Stacked column graphs of percentage of cases that are either cohesive or non-cohesive. Scale bars: 300 μm.

As the LaNt α31 functional studies data suggested that high expression of this protein changes the mode of tumour invasion, we returned to the tissue array data, focusing specifically on the cores with high LaNt α31 intensity and assessed the tumour appearance in each of those cores as either “cohesive” or “non-cohesive” depending on whether tumour cells were present in contiguous islands with well-defined borders, (representative examples Fig 10A). These analyses revealed that 67.7% of the high LaNt α31 expressing tumours were non-cohesive in appearance (21 of 31 cores, Fig 10B).

## Discussion

The findings presented here have revealed that the little-known LM-related protein LaNt α31 is upregulated in a distinct sub-population of breast cancers, including a subset of TNBC, and correlates with tumour cohesiveness but is not predictive of nodal involvement. In vitro studies revealed that LaNt α31 expression was not sufficient to confer invasive nor increased migratory capabilities into a non-invasive breast cancer cell line; however, LaNt α31 expression converted the mode of invasion of an already invasive line when invading into a LM-rich matrix. These findings are consistent with a potential effect of LaNt α31 upon tumour cohesiveness and suggest that dysregulation of this protein could actively contribute to defining how breast cancer cells disseminate.

Usually in tumour situations, very few cells acquire the ability to invade and a hallmark of more aggressive tumours is plasticity in the modes of migration [46, 47]. A switch from multicellular streaming to individual cell invasion can happen in multiple overlapping ways. A major driver is the mechanical properties of the ECM including matrix stiffness and the orientation of fibres; however decreased cell-cell adhesion, increased Rac-driven cytoskeletal protrusion compared with Rho-mediated contraction, an increased ability to generate traction forces, and differences in proteolytic activities all can drive the changes [44], as reviewed in [48]. Many of these mechanisms are intrinsically linked, which makes it challenging to directly assign a single behaviour to an individual protein. However, the Matrigel-specific effects and the structural similarity between netrin-4 and LaNt α31 make it tempting to predict that LaNt α31 has a disruptive effect on LM networks, softening or disordering the matrix through which the cells are invading and providing a permissive environment for tumour metastasis [19, 21].

The differences in responses between MCF7 and MDA-MB-231 could also provide support for a matrix-disruptive mechanism. MDA-MB-231 are more mesenchymal-like compared with the more epithelial-like MCF7, and this is associated with their intrinsic differences in migration behaviours; individual cell migration and multicellular streaming for MDA-MB-231, versus collective for MCF7. The invasive MDA-MB-231 line when cultured on Matrigel has also been reported to express much higher levels of MMP9 than MCF7s, and this expression is required for their invasive capabilities [49]. LN domains, including the LN domain of LMα3b, are known to have cell-surface receptor-binding capabilities [50] [51]. Notably, netrin-4 and a proteolytically released LN domain fragment of LMβ1 are each capable of inducing epithelial to mesenchymal transition and have been shown to regulate expression of MMPs [19, 20, 52, 53]. Therefore, the LaNt α31 effect could be an indirect consequence of cell receptor activation through a similar but as yet unidentified mechanism. Matrigel also contains many components beyond LM111 [43], therefore the observed invasion phenotype could be due to LaNt α31 interaction with addition non-LM factors. Dissecting the mechanism of the LaNt α31-induced changes will not be a trivial undertaking but will be valuable to understand the processes involved. Moreover, in vivo analyses will be required to determine the contribution to tumour progression of LaNt α31 and to identify any cause-and-effect relationship between LaNt α31 expression and tumour cohesiveness, and the importance of these effects to tumour progression.

One finding that has been hard to reconcile is despite the association between high LaNt α31 expression and cancer and increased LaNt α31 expression in metastasis relative to primary tumours, there were no statistically robust associations between LaNt α31 expression level and nodal involvement or patient survival. This collection of findings is somewhat counter-intuitive and while they may be indicative of a true lack of effect of LaNt α31 on patient outcome, the data may also point to more complex subtype effects that are not captured by our limited molecular phenotyping data and by sample sizes which were not amenable to further sub-group analyses. Subsequent analyses may be warranted with stratification on markers such as EGFR, as this could provide indications of mechanism. It may also now be valuable to now assess co-distribution patterns of putative LaNt, netrin and laminin receptors.

Secondly, while the tissue data immunohistochemistry results indicated that tumours with high Ki67 percentage were more likely to display medium to high LaNt α31 immunoreactivity, stratification by proliferation status of the underlying tumour did not improve predictions of nodal involvement. Moreover, the in vitro studies indicated that LaNt α31 expression is not dependent on proliferation, and that induced expression reduced rather than increased cell numbers both in MCF7 and in MDA-MB-231 cells in culture. Together these findings could suggest that the Ki67/ LaNt α31 expression could be a spurious correlation. However, it should be noted that the in vitro work only involved epithelial-derived tumour cells cultured on solid 2-D substrates and does not account for the complex tumour environment. The ostensibly disparate findings in tumour sections compared with cultured cells may reflect the LaNt α31 protein acting on the matrix, influencing matrix stiffness, pore size or signal propagation, and thereby LaNt α31 may indirectly influence cell cycle progression when in its true biological context. This is a complex question, requiring advanced in vivo models to address, and will be the focus of future work.

An additional intriguing finding was the difference between LaNt α31 and LMα3. Although these proteins are genetically linked, they are structurally and functionally distinct. Specifically, LMα3a, as part of LM332, has been robustly demonstrated to enhance the migratory behaviour of MCF7 and MDA-MB-231 cells in culture [54, 55]. However, loss or focal disruption of LM332 staining is a more common feature in breast cancer, particularly for LMα3b, which shares a promoter with LaNt α31, and is downregulated in the tumour vasculature [26]. Indeed, in side-by-side comparison of the same tissue, the observation of different structures displaying upregulation of LaNt α31 compared with LMα3 points to differences in post-transcriptional regulation. We do not yet know if the difference is due to differences between the isoforms in terms of pre-mRNA processing, mRNA degradation, or post-translational proteolytic processing; however, these data do suggest that changes to LaNt α31 expression may have more widespread implications to other cancer subtypes where LMα3 is known to be dysregulated. In these contexts, processing tissue for LaNt α31 may have value as a biomarker.

## Conclusions

The combination of patient and experimental data presented here have revealed for the first time that LaNt α31 is associated with breast cancer. Importantly, that LaNt α31 influences the mechanisms through which invasive breast cancer cells migrate in a 3-D environment. These findings suggest that LaNt α31 contributes to how a tumour progresses and raises the potential that targeting this protein’s function could hold therapeutic value.

## Supporting information

S1 Fig LaNt α31 is upregulated in ductal carcinoma and in lymph node metastases. Formalin-fixed paraffin-embedded human breast tissue microarray sections processed for immunohistochemistry with mouse monoclonal antibodies against LaNt α31. Two separate arrays were used; (A) uninvolved with paired invasive/ in situ ductal carcinoma tissues (N = 25), (B) invasive ductal carcinoma with paired node metastases (N = 29). All paired cores analysed in Fig 2 ordered based on LaNt α31 staining intensity, low to high (left to right). Scale bars: 500 μm.
(TIF)

S2 Fig Proliferation is not required for LaNt α31 expression. MCF-7 or MDA-MB-231 cells were treated with 10 ug mL^-1^ mitomycin c for either 2 h or left with the drug overnight. (A) Hoechst 33342 was added to the culture media, and the cell nuclei imaged after 20 min. (B) After 24 h, total RNA was extracted and one-step RT-qPCR performed to quantify LAMA3LN1 transcript abundance, normalising to GAPDH and RPLP0 reference transcripts.
(TIF)

S3 Fig Uncropped immunoblot and qRT-PCR melt curves. (TIF)

S1 Movie MDA-MB-231 +eGFP invasion into Matrigel. MDA-MB-231 cells transduced with eGFP were plated onto the outside of a transwell membrane. 10 ng mL^-1^ epidermal growth factor was used to stimulate invasion through the membrane and into Matrigel. After 48 h, the cells were fixed and stained with DAPI then imaged at 5 μm intervals using a spinning disk confocal microscope. Representative movie of invasion profile generated from z-stack slices for invasion between 20 and 60 μm after applying threshold.
(AVI)

S2 Movie MDA-MB-231 +LaNt α31-eGFP invasion into Matrigel. MDA-MB-231 cells transduced with LaNt α31-eGFP were plated onto the outside of a transwell membrane. 10 ng mL^**-1**^ epidermal growth factor was used to stimulate invasion through the membrane and into Matrigel. After 48 h, the cells were fixed and stained with DAPI then imaged at 5 μm intervals using a spinning disk confocal microscope. Representative movie of invasion profile generated from z-stack slices for invasion between 20 and 60 μm after applying threshold.
(AVI)

S1 Raw images (TIF)

## Abbreviations

BM: Basement membrane
ECM: Extracellular matrix
EGF: Epidermal growth factor
eGFP: Enhanced green fluorescent protein
EGFR: Epidermal growth factor receptor
ER: Estrogen receptor
FFPE: Paraffin-fixed formalin-embedded
Her2: Human epidermal growth factor receptor 2
Ki67: Antigen ki-67
LaNt α31: Laminin N terminal protein α31
LE-repeat: Laminin-type epidermal growth factor-like
LM: Laminin
LN domain: Laminin N-terminal
p53: Tumour protein p53
PR: Progesterone receptor
RT-qPCR: Reverse Transcription-quantitative polymerase chain reaction
TDLU: Terminal duct lobular unit
TNBC: Triple negative breast cancer
