# Supplementary figures and images for: Laminin N-terminus α31 is upregulated in invasive ductal breast cancer and changes the mode of tumour invasion
Source: PLoS One. 2022 Mar 1;17(3):e0264430. doi: 10.1371/journal.pone.0264430 (PMC8887744; doi:10.1371/journal.pone.0264430)

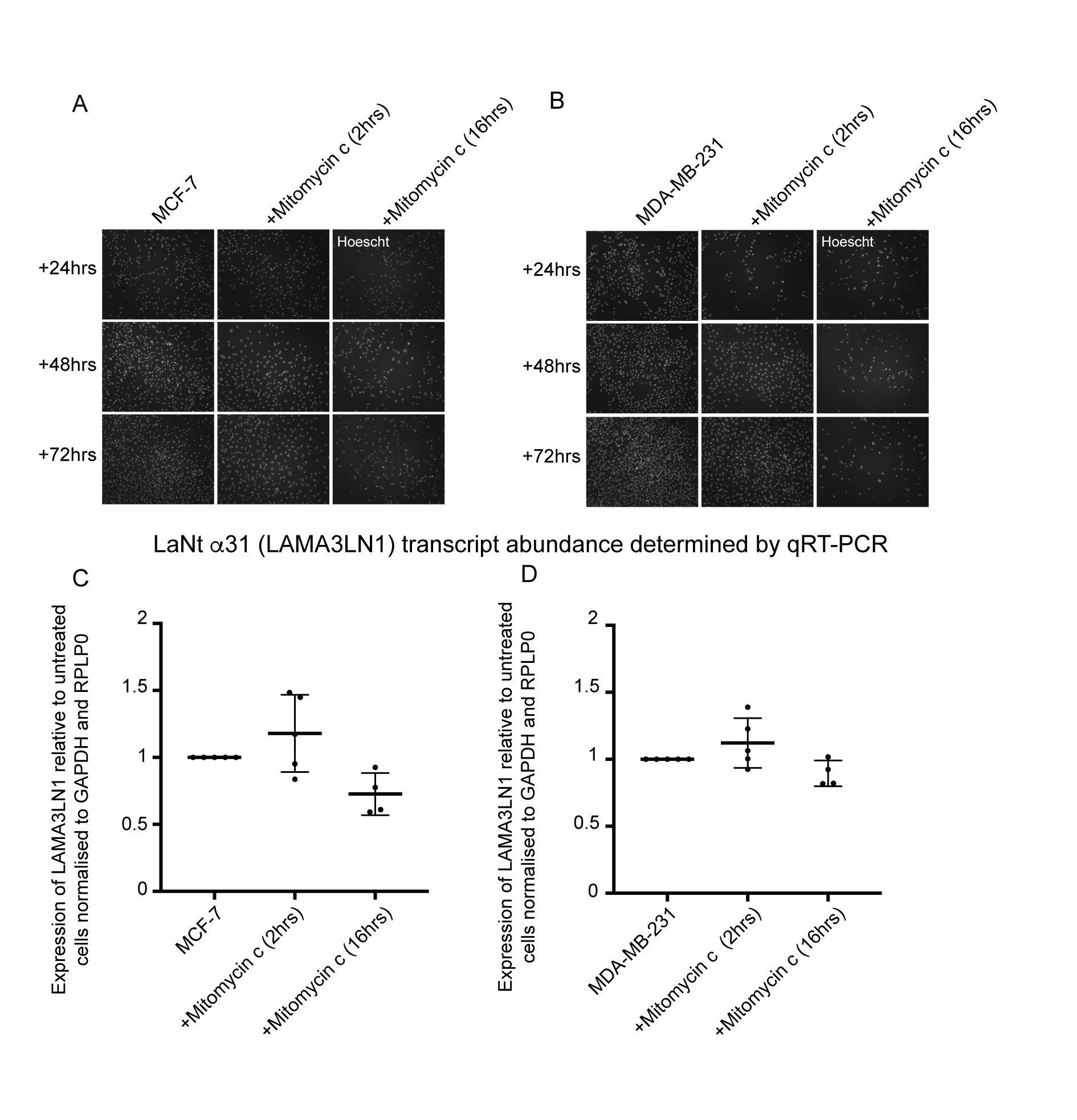

Supplement: S2 Fig — MCF-7 or MDA-MB-231 cells were treated with 10 ug mL-1 mitomycin c for either 2 h or left with the drug overnight. (A) Hoechst 33342 was added to the culture media, and the cell nuclei imaged after 20 min. (B) After 24 h, total RNA was extracted and one-step RT-qPCR performed to quantify LAMA3LN1 transcript abundance, normalising to GAPDH and RPLP0 reference transcripts. (TIF) [file pone.0264430.s002.tif]

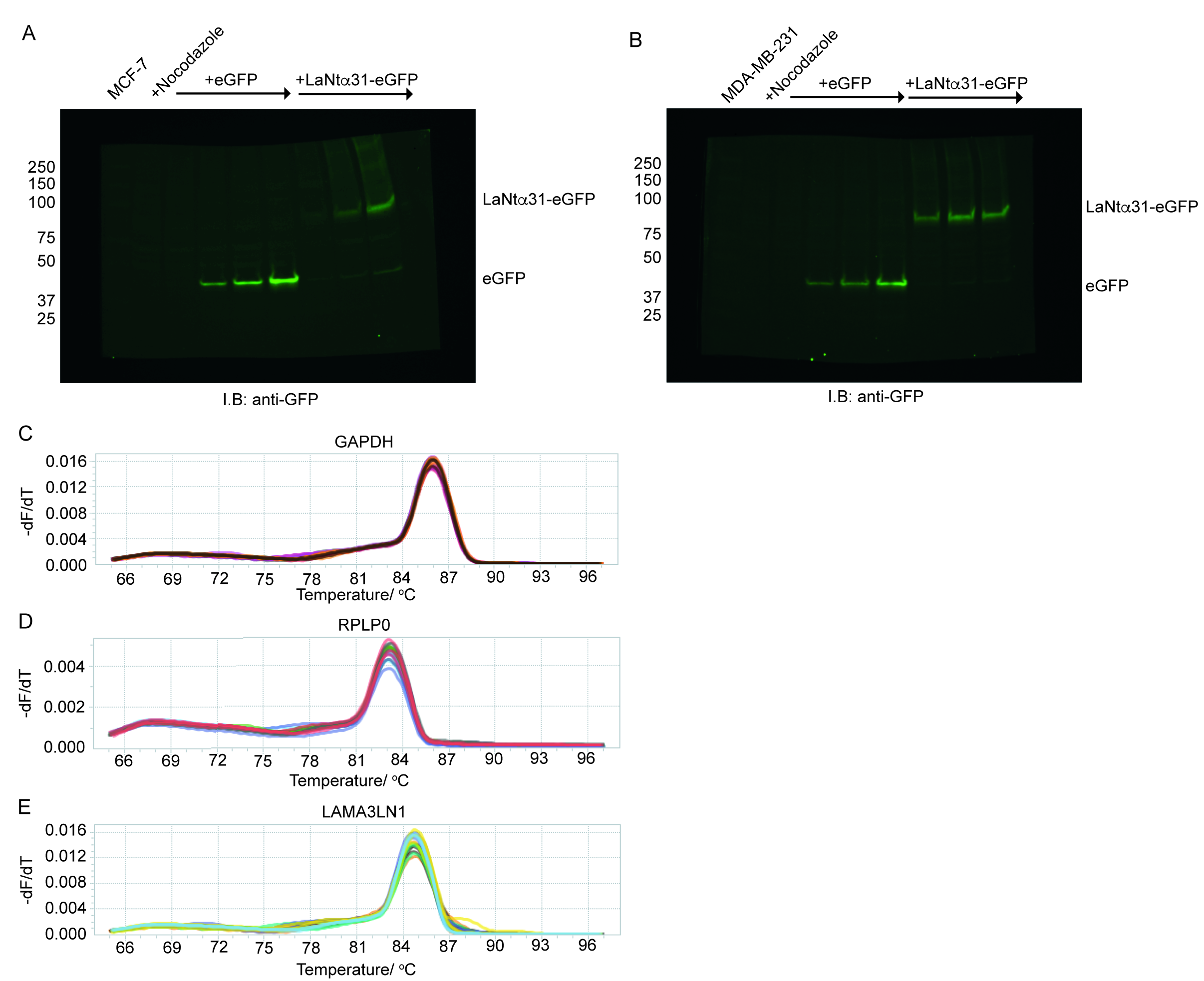

Supplement: S3 Fig — (TIF) [file pone.0264430.s003.tif]

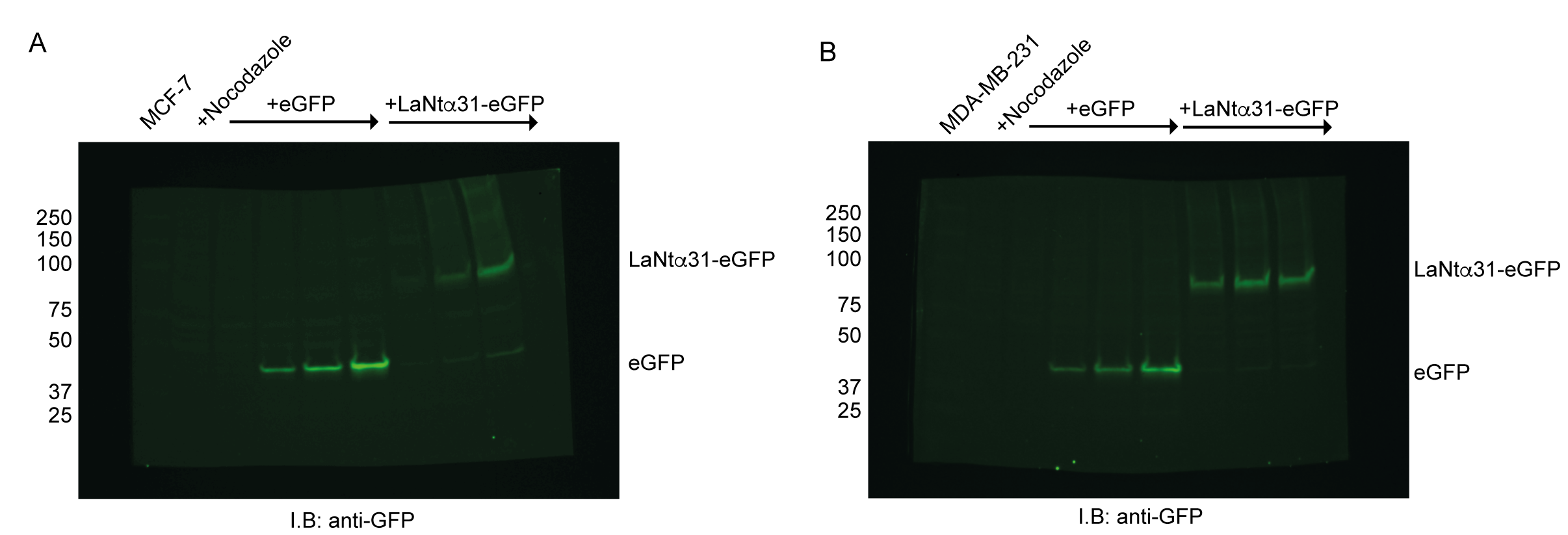

Supplement: S1 Raw images — (TIF) [file pone.0264430.s006.tif]
